# Energy Efficient In-network RFID Data Filtering Scheme in Wireless Sensor Networks

**DOI:** 10.3390/s110707004

**Published:** 2011-07-06

**Authors:** Ali Kashif Bashir, Se-Jung Lim, Chauhdary Sajjad Hussain, Myong-Soon Park

**Affiliations:** Department of Computer and Radio Communication Engineering, Korea University, Seoul 136-713, Korea; E-Mails: alik@korea.ac.kr (A.K.B.); limsejung@korea.ac.kr (S.-J.L.); Sajjad@korea.ac.kr (C.S.H.)

**Keywords:** RFID data filtering, energy-efficiency in WSN, in-network data processing, integration of RFID with WSN, redundant readers

## Abstract

RFID (Radio frequency identification) and wireless sensor networks are backbone technologies for pervasive environments. In integration of RFID and WSN, RFID data uses WSN protocols for multi-hop communications. Energy is a critical issue in WSNs; however, RFID data contains a lot of duplication. These duplications can be eliminated at the base station, but unnecessary transmissions of duplicate data within the network still occurs, which consumes nodes’ energy and affects network lifetime. In this paper, we propose an in-network RFID data filtering scheme that efficiently eliminates the duplicate data. For this we use a clustering mechanism where cluster heads eliminate duplicate data and forward filtered data towards the base station. Simulation results prove that our approach saves considerable amounts of energy in terms of communication and computational cost, compared to existing filtering schemes.

## 1. Introduction

The next revolution in computing technology is the widespread use of small wireless computing and communication devices that will integrate seamlessly into our daily life [1,2]. Therefore, in the near future we can expect the use of lots of devices such as tags, sensors, and readers *etc.* to grow by many orders of magnitude. From a technology perspective, RFID and sensor networks are important components of this paradigm, since both technologies can be used for coupling the physical and virtual worlds, usually known as pervasive computing [3].

WSNs are networks of small, cost effective devices with sensing, data processing, and communication ability. WSN are being used for several applications ranging from military surveillance to habitat monitoring. In these applications, WSN are just sensing the environment and sending data to a base station. Therefore, they are not providing any contextual information. However, integrating the WSN with RFID provides context to the sensed data. This integration has facilitated our lives in many areas such as supply chain management [4], health care [5], tracking and monitoring of objects and humans [5,6].

**Figure 1.**
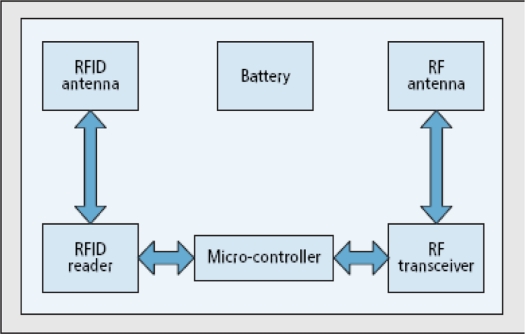
Integrated WSN node and RFID Reader.

RFID technology was developed to replace traditional barcode systems. It consists of reader, tags, and applications. Readers read the tags attached on objects, store data in their memory, and the applications access it. Existing RFID technology does not support multi-hop communication from reader to reader. By integrating it with WSN, we can route RFID data from readers to base stations/servers/applications by using existing sensor network protocols. For this, nodes can have both functionalities: sensing and reading, as shown in Figure 1. There are several other ways of integrating RFID with WSN [3,7,8].

On the other hand, RFID data is unreliable by nature and usually the observed read rate of a reader (*i.e*., number of tags read to the actual number of tags) is 60–70% and 30–40% is the missing ratio [9]. To increase the accuracy of read data, readers interrogate tags periodically. These multiple readings resolve the poor reading rate problem; however, it generates a lot of duplications by reading already read tags multiple times. Moreover, in WSNs the nodes are densely deployed and have overlapping areas with neighboring nodes. Tags that exist in overlapping areas are read by more than one reader which results in duplicate data generation. Transmitting these duplicate data packets towards the base station consumes enormous amount of node energy, whereas, energy consumption is an important issue in WSNs due to the limited battery life of the nodes. Duplication can occur in many ways. However, generally they can be divided into three categories, as given below:

- *Multiple Read Cycle:* Tags in the vicinity of a reader for a long time (in multiple reading cycles) are read multiple times [9].
- *Redundant Reader:* Multiple readers are installed to cover larger area, and tags in the overlapped areas are read by multiple readers [10].
- *Data level:* Multiple tags with same EPC (Electronic Product Code) are attached to the same object in order to reduce missing rate and increase reliability [11].

Many researchers have proposed schemes to filter duplicate data at the application server [9,12]. However, transmitting these redundant packets will affect the nodes’ energy and result in transmission overhead and decreased network lifetime. To avoid these unnecessary transmissions, redundant data should be processed within the network. In [13,14] authors proposed to reduce the transmission overhead by performing in-network processing in the WSN. In-network processing saves considerable amount of nodes’ energy. On the other hand, processing all the data within the network increases computation overhead and induce delays. Kadayif *et al*. [15] discuss the trade-off between communication and computation cost in sensor network applications. However, these approaches only deal with sensor data where aggregation of data is possible. RFID data cannot be aggregated as every tag has its own identity, but due to enormous amount of duplication, we can filter this data within the network to avoid redundant transmissions.

*In-network phased filtering mechanism* (INPFM) [16], and *Cluster-Based In-Network Phase Filtering Scheme* (CLIF) [17] filter RFID duplicate data within the network; however, these approaches have high computation costs and they do not reduce much the transmission overhead. In this paper, we introduce *Energy-Efficient In-Network RFID Data Filtering Scheme* (EIFS). It exploits the clustering topology and divides the duplication into two phases: intra-cluster duplications and inter-cluster duplications. We discuss these duplications separately and provide algorithms for each. We have conducted simulation in C and compared our approach with INPFM and CLIF. As the simulation results show, EIFS saves a considerable amount of transmission overhead by filtering redundant data. Moreover, the computation cost is much lesser compared to other two schemes.

The rest of the paper is organized as follows: Section 2 contains the related works. In Section 3, we discuss the problem formulation, system model, data model to filter data, and a node distinction algorithm. In Section 4, we presented our proposed EIFS scheme that contains two different algorithms for duplicate detections: intra-cluster duplication is discussed in Section 4.1 and inter-cluster in Section 4.2. In Section 5 we discussed our simulation results and lastly our conclusions are presented in Section 6.

## 2. Related Work

Data filtering is as an important issue in RFID applications. Enterprises/applications are interested in single copies of data. In last few years, several researchers have provided solutions for filtering RFID data. The authors of [9,12] proposed their approaches to filter duplicate data using a sliding-window. The sliding window keeps the history of the previous read cycles in a buffer and outputs the data when it increases above a certain threshold. These approaches filter considerable amounts of data and also remove other anomalies such as noise from data. However, deciding the appropriate size of sliding window is still an open research question. Moreover, these solutions are proposed for middleware at the base station. This middleware can be implemented within the readers, but due to limited memory of readers this is not an appropriate solution. On the other hand, filtering redundant data at the base station does not decrease the transmission overhead at the nodes, so we need to process data within the network to remove duplications.

In-network processing in WSNs is being researched intensively in terms of data aggregation [18–22] and data fusions techniques [23,24]. In typical sensor network scenarios, data is collected by sensor nodes throughout some area, and needs to be made available at some central sink, where it is processed, analyzed, and used by the application. In many cases, data generated by different sensors can be jointly processed while being forwarded towards the sink, e.g., by fusing together sensor readings related to the same event or physical quantity, or by locally processing raw data before this are transmitted. In-network aggregation deals with this distributed processing of data within the network. Data aggregation techniques are tightly coupled with how data is gathered at the sensor nodes as well as how packets are routed through the network, and have a significant impact on energy consumption and overall network efficiency (e.g., by reducing the number of transmissions or the length of the packets to be transmitted).

These techniques reduce transmission overhead, but on the other hand, they also increase computation overhead at the nodes. Therefore, it is required to maintain a balance between communication and computation costs to meet the desired objectives of applications. Kadayif [15] proposed such a strategy to maintain a balance between computation energy and communication energy in wireless sensor networks. This approach transfers the code that reduces the output size of data packets from base station to sensor nodes. Moreover, sensor nodes decide whether output data needs to be forwarded to the base station or not. If the output data is used for further processing, it will be processed at the node and a smaller number of outputs will be sent to base stations. This approach provides a trade-off between computation and communication energy.

In previous research, in-network filtering in RFID applications has been studied as a duplicate data filtering or noise removal issue. However, the objective of this study is only duplicate data elimination. In-network filtering in RFID has not been widely researched; therefore only few studies exist. Carbunar *et al*. [10] resolved the problem of redundant readers, where readers are overlapped and produce duplicate readings. They resolve this problem by temporarily deactivating the readers that have maximum overlapped region with neighboring readers. For this, Carbunar determined the minimal subset of the readers that can cover the whole area. This mechanism reduces the redundant transmission, but in large deployments finding which readers need to be turned off is an NP-hard problem [25]. To filter data level and multiple read cycle duplications, a few simpler solutions have been provided [9,11]; they design algorithms to filter duplicate data at the reader level. Every reader filters only its own data and forwards non-duplicated data towards the sink. However, a real challenge occurs when readers have to filter duplicate data generated due to overlapping.

Wonil *et al.* [16] proposed INPFM that filters duplicate data at every *k* hop reader where *k* varies according to duplication ratio of tags. INPFM claims that filtering data at every hop induces delays. This approach follows a tree structure with multi-hop routing and parent nodes filter child nodes duplicate data if they detect it. However, in dense deployments where duplication is enormous, duplicate data might not meet at a filtering point. Moreover, although data is filtered at *k* hope distance, the duplicate detection mechanism works on every node and results in a huge computational overhead. To filter data closer to the origin, Dongsub *et al*. [17] exploit the clustering topology and divide duplication into intra-cluster and inter-cluster. Intra-cluster duplication is filtered at a local CH and inter-cluster duplication at distant CHs. CLIF improves the performance compared to [17], but it also has high computation overhead as their inter-cluster data filtering approach is similar to that of INPFM.

## 3. Preliminaries

### 3.1. Problem Formulation

**Figure 2.**
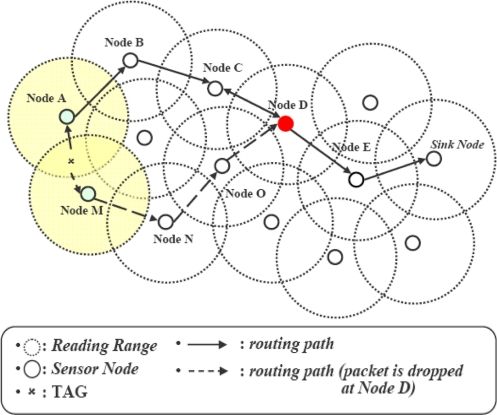
Filtering the data at a distant point.

Duplicate readings generated by overlapped readers result in unnecessary transmissions. This consumes network bandwidth and decreases the network lifetime. The proposals in [16] and [17] eliminate the duplication during the transmission phase as shown in Figure 2. INPFM [16] follows the tree structure whereas CLIF [17] follows the clustering approach and filters the inter-cluster duplication at some intermediate CH. In Figure 2 nodes “A” and “M” interrogate tags in overlapping areas, such as *x* in this example, and transmit data to the sink node by multihop routing. INPFM filters this data at *k* hop distance. In CLIF, if these two nodes are part of same cluster, this duplication will be filtered at a local CH; if these two nodes are from different clusters, then this might be filtered at an intermediate CH.

These approaches save the transmission overhead by filtering duplications. However, several problems still exist. First, the duplicate detection module runs on every node or CH for all arriving data. This increases the computation cost. Second, although duplicate data is filtered at a CH in first round, duplicate transmissions from the source node to the filtering point happens for every subsequent round. Such transmission is unnecessary but it continues as long as the tag is in overlapped area. Moreover, performance of these approaches degrades with increased number of tags in overlapping regions, resulting in increased computation cost at nodes and inducing delays.

To resolve these problems, we proposed the Energy Efficient RFID Data Filtering Scheme (EIFS) filtering scheme. Our scheme exploits the clustering topology like CLIF [17]; however, comparatively it filters data close to the origin of duplicate data generation to avoid redundant transmission. Moreover, the computational cost of our algorithm is much better than those of the existing algorithms.

### 3.2. System Model

In this paper, we consider a sensor network consisting of *N* sensor nodes deployed densely to cover the whole area. All nodes are homogenous in nature and are grouped into clusters. One node is part of one cluster. The cluster head task can be rotated on a probability basis to balance the energy consumption among nodes, but this is not within the scope of our work. Every node has sensing and reading module as shown in Figure 1. They read tags in multiple cycles. The following are the assumptions of our environment:

- Nodes are formed into clusters and they can communicate with the CHs directly.
- RFID readers read the tags in multiple frames and forward data to cluster heads.
- Only cluster heads will execute the filtering algorithm and will forward filtered data along the routing path towards the base station using multi hop communication.
- Nodes are homogenous and static in nature. Their transmission range is double the reading range.

### 3.3. Node Distinguishing Mechanism

**Figure 3.**
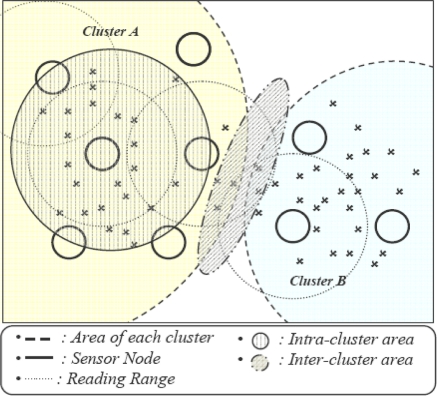
Redundancy definition.

The density of the WSN results in overlapping of nodes and tags in overlapping regions are read by more than one reader, which results in duplicate data generation. This duplication grows with the density of the network. In clustering topology, duplication can be divided into the following two types as shown in Figure 3.

*Intra-cluster duplication:* Nodes having overlapping areas with neighboring nodes within a cluster are called intra-cluster nodes. All the nodes of a cluster send data to their own cluster head, and after eliminating duplication, the cluster head routes data towards the base station. This filtering procedure reduces redundant data transmission within the network.

*Inter-cluster duplication:* Nodes overlapped with neighboring cluster nodes are called inter-cluster nodes and such duplication is called as inter-cluster duplication. Such duplication can be filtered at intermediate cluster heads as proposed in [17], but transmission of duplicate data from origin to filtering point that results in transmission overhead still occurs. To avoid this overhead, EIFS filters this data at neighboring CHs.

**Figure 4.**
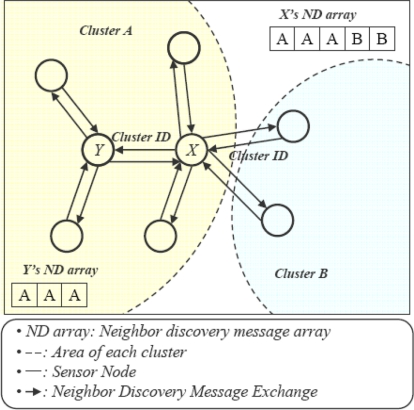
Node distinguish mechanism (decision about inter-cluster or intra-cluster duplication).

Inter-cluster duplication can’t be detected by a single CH without exchanging information with neighboring CHs which results in a huge communication overhead. We have provided two different mechanisms to detect and filter intra and inter-cluster duplications. But, first we need to distinguish among readers that overlap within clusters or across the boundary of a cluster, for this, we have introduced the Neighbor Discovery Message (*ND)* as shown in Figure 4.

In our approach after cluster formation, each node exchanges an *ND* message with neighboring nodes. The ND message contains node *ID* and cluster *ID*. A node that receives *ND* messages from its neighbors keeps the cluster *ID* in an *ND* array. From the *ND* array of a node, we can know whether it has the *ID* of any neighboring clusters or not. If *IDs* of two or more than two clusters exist in a node *ND* array that node will be considered as inter-cluster node. However, at the same time one node can have duplication with nodes of the same cluster and with nodes of different clusters.

### 3.4. RFID Data Modeling

Table 1 describes the structure of the RFID data packet; tag *ID* represents the identification of the tag. In this paper, EPC GID-96 is used for the tag *ID* since it is the most popular type in current commercial RFID systems. Reader *ID* represents the address of the reader. For filtering, we assign two kind of initial values to *number of* remaining *filtering*, 1 and *f_e_*. In case of intra-cluster nodes, value of *f* will be 1. Whereas, in inter-cluster node value will be *f_e_* as shown below:

$$\begin{array}{l} \mathrm{Number of remaining filtering}\;(f)=1:\;\mathrm{need to be filtered at local CH}.\\ \;\mathrm{OR fe}:\;\mathrm{need to be filtered at intermediate CH}. \end{array}$$

When a cluster head receives data packets from its cluster members, it checks from the tag list whether the incoming RFID data packet is already received or not. The structure of the tag list is shown in Table 2. The observation *(β)* field has two flags such as *R* and *D*. *R* means that the RFID packet is successfully relayed to the sink node and *D* represents that the RFID packet is dropped for duplication at an intermediate node. Redundant reader *ID (N)* indicates the reader that reads the tag and generates the intra-cluster duplications. If tag *ID*, reader *ID*, and time stamp all match; and value of *β* is as ‘*D*’ and *N* exists, it means this data is already dropped at previous readings.

## 4. EIFS: Energy Efficient In-Network RFID Data Filtering Scheme

An efficient in-network RFID data filtering scheme should filter the maximum amount of data to avoid redundant transmission in the network with less computation. To meet these objectives, EIFS divides the duplications into two types. We address each of them separately in the following sections. First we would like to present the structure of data packets in the inter-cluster and intra-cluster cases.

### 4.1. RFID Data Packet Generation/Data Transfer Phase

The type of a node can be known from the *ND* array. When an intra-cluster node, suppose *n*, interrogates a tag *x*, after tag response, the node generates an RFID data packet with the value of *f* (number of remaining filtering operations) as 1, shown in Table 3. The node sends this data packet to its cluster head. If any neighboring node also report to cluster head with tag *x*, the cluster head will filter it to avoid redundant transmission in the network. In the case of inter-cluster nodes, the value of *f* is *f_e_*. Initial value of *f_e_*, is the system parameter. Table 4 shows the structure of the RFID data packet in inter-cluster node. Every node sends their data to its cluster heads and they decide the type of sender from the *f* field. If the value of *f* is 2 or more than 2, the sender is considered an inter-cluster node. Otherwise, it is intra-cluster node.

### 4.2. Intra-Cluster Filtering

**Figure 5.**
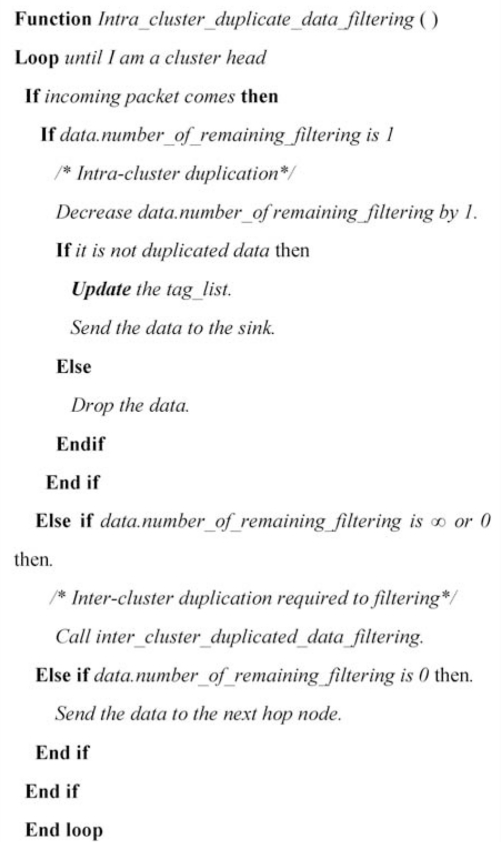
Intra-cluster filtering algorithm.

When a cluster head receives an RFID data packet, it decides the type of sender by the *f* field. If the value of *f* is 1, the sender is an intra-cluster node and the cluster head need to execute the filtering algorithm to check the duplication. After removing the duplication, it sets the *f* field as 0 and forwards the data towards base station. Such a packet will not be filtered on any intermediate cluster head which saves computation costs in comparison with INPFM where the filtering process runs for every arriving packet. For an intermediate CH when the value of *f* is 0, it means data is coming from an inter-cluster node from another cluster and it is already filtered data. This mechanism significantly reduces the number of comparisons. Conditions of filtering and relaying are given below and algorithm is given in Figure 5.

(1) $$\begin{array}{l} \text{The condition of perform a filtering}:\\ \;\mathrm{perform}=\{\begin{array}{lll} \mathrm{true}, & \mathrm{if} & f\geq 1\\ \mathrm{false}, & \mathrm{if} & f=0 \end{array} \end{array}$$

(2) $$\begin{array}{l} \text{The condition of date relay}:\\ \;\mathrm{relay}=\{\begin{array}{lll} \mathrm{true}, & \mathrm{if} & f=0\\ \mathrm{false}, & \mathrm{if} & \mathrm{the data is duplicate} \end{array} \end{array}$$

### 4.3. Inter-Cluster Node Filtering Algorithm

After intra-cluster duplications, CHs send their data towards sink along the route. Intermediate CHs will detect both intra-cluster duplication of its own member nodes and inter-cluster duplication between its own data and also data coming from other CHs. In the literature, several routing schemes have been proposed to improve performance and save energy such as shortest path tree [26], greedy [27] or geographical routing [28]. The performance of these protocols will vary with the environment and applications of the RFIDs. However, in this work, we have not evaluated the performance of our algorithm with these routing schemes. After inter-cluster duplicate detection, intermediate CHs will inform with a feedback message to CHs whose nodes are generating duplicate data packets. Later, those CHs can change routing paths of duplicate data to eliminate it close to source, at neighboring CHs, to avoid redundant transmissions from data generation point to detection point, whereas, in INPFM [16] and CLIF [17] such duplicate transmissions happen in every round. However, all these schemes assume tags are not mobile or their mobility is sparse. Our detailed algorithm is presented below.

#### 4.3.1. Feedback Message

**Figure 6.**
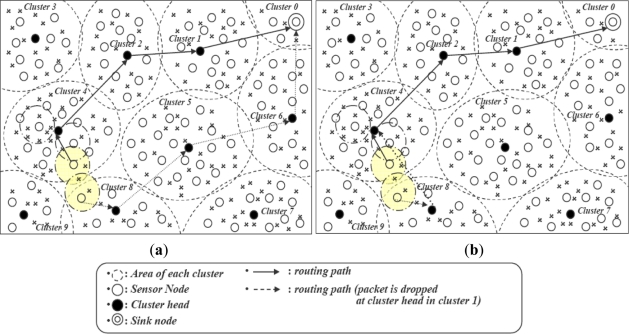
Inter-cluster duplicate detection and elimination: (**a**) Duplicate data elimination at sink; (**b**) Modification of routing path or intermediate node.

In dense deployments, the ratio of duplicate data generation increases with the network size and number of clusters. Our proposed algorithm EIFS first detects the inter-cluster duplicate data at intermediate nodes and sends a feedback message. In Figure 6(a), an intermediate CH detects the inter-cluster duplication of CHs 4 and 8. After the filtering process, late arrival data will be dropped and the source CH informed about the duplication with a *feedback message*. The source CH modifies its routing paths of duplicate data towards the CH with which inter-cluster duplication is happening as shown in Figure 6(b).

By this, we can significantly reduce unnecessary transmissions. Our approach changes the routing path of late arrival data at an intermediate CH, in simple words, the late arrival data might have longer route or have some delay en route to an intermediate CH. Changing routing paths of duplicate data packets will help in balancing delay and energy on routing paths. However, this is not the objective of our current work.

*Feedback message* includes tag *ID* and reader *ID* in tag list. Once a cluster head receives the feedback message, it checks tag *ID* from its tag list to update the observation field as *D* which means tag is dropped at intermediate CH due to duplication, and *N* as reader *ID* that have overlapping region with nodes of neighboring cluster according to structure of inter-cluster node presented in Table 4. CH uses these values in next round for minimizing the transmission overhead. However, huge inter-cluster data will result in a lot of feedback messages. To avoid frequent feedback messages we set a condition. If the following condition is true, the intermediate CH will send a feedback message, otherwise it discards the feedback message.

(3) $$\begin{array}{l} \text{The condition of sending Feedback Message}\\ \;\mathrm{sending}=\{\begin{array}{ll} \mathrm{true}, & f<f_{e}-\alpha \\ \mathrm{false}, & f\geq f_{e}-\alpha \end{array} \end{array}$$

where *f_e_* is the initial value of *f* in inter-cluster node and *“α”* means *minimum number of filtering processes to not send the feedback message to the source CH*. For example, if the tags are dropped at neighboring CH, the f*eedback message* is not needed. The source CH will send data to the BS along the previous route. However, it might possible that route is already changed by feedback message in a previous detection. This procedure will save a lot of communication overhead. Measuring the exact value “*α*” depends on the network size, number of cluster, or distance of a node from base station.

### 4.4. Data Filtering Phase

In the previous section, we mentioned that if an intermediate CH detects duplication, it informs the CH whose data arrives later with a feedback message. The sender CH receives that message and updates the tag list of tags that are involved inter-cluster duplication.

**Figure 7.**
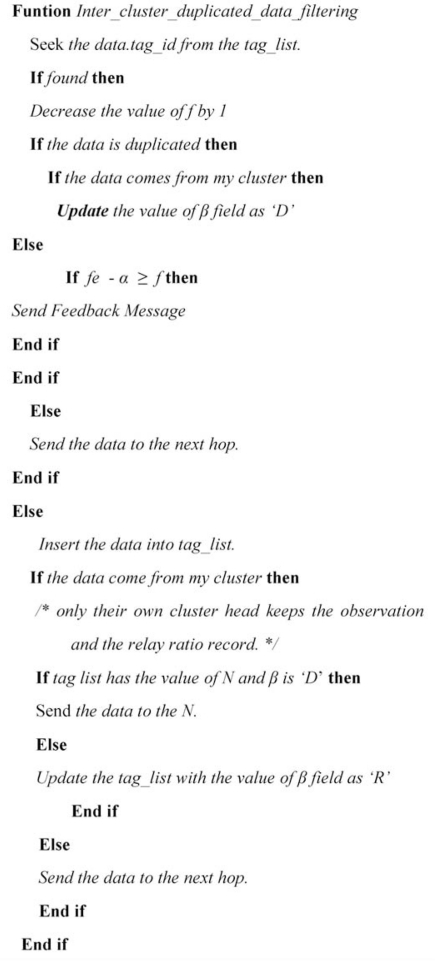
Inter-cluster filtering algorithm.

In the next round, when a CH receives data from inter-cluster nodes, it checks its tag list. If there were a feedback message against some tag data, it modifies the routing path of these tags towards neighboring CH with which node inter-cluster duplication is occurring. By this, inter-cluster data is being dropped at neighboring CH’s instead of intermediate CH’s. Dongsub *et al.* [17] filter this data at intermediate CH’s. Feedback messages also consume energy; however, for an environment in which tag mobility is sparse and deployment is dense, filtering data close to the source saves a considerable amount of redundant duplicate transmissions. Our detailed algorithm is given in Figure 7.

## 5. Simulation Results

In our previous sections, we claimed that EIFS filters duplication efficiently while saving energy in terms of computation and communication costs. In this section, we will compare the performance of our algorithm with INPFM [16] and CLIF [17]. INPFM uses a tree structure and it filters data at every *k* hop node from source; the authors claim that filtering all the data within the network increases computation overhead and causes delays, whereas CLIF uses a clustering approach and divides duplication in intra-cluster and inter-cluster. Clustering is more efficient in terms of in-network processing. In our approach, we also exploit the clustering topology; however, compared to CLIF our approach filters the inter-cluster duplication close to the source and saves considerable communication cost. We have developed a simulator using C++ in which nodes are densely deployed and exist in the shape of clusters. We define the duplication as *two or more than two readings having the same tag id with a time difference of less than 2 seconds*. The detailed simulation environment is given in Table 5.

**Figure 8.**
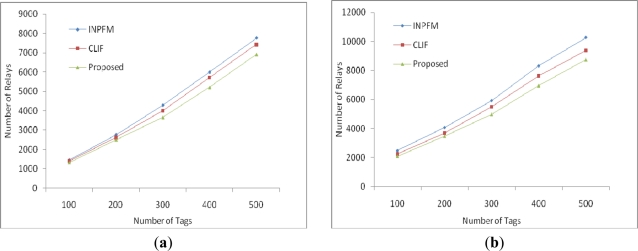
Communication Cost in terms of Reduced Number of Transmissions (**a**) Sparsely disseminated tags with duplicate ratio 20%; (**b**) Densely disseminated tags with duplicate ratio 50%.

Firstly, we calculate communication cost in terms of number of relays required to disseminate tag readings towards the base station. EIFS performs better than INPFM and CLIF as shown in Figure 8.

**Figure 9.**
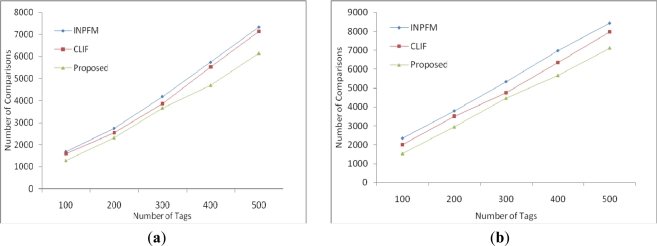
Computational cost in terms of number of comparisons required to filter data: (**a**) Sparsely disseminated tags with duplicate ratio 20%; (**b**) Densely disseminated tags with duplicate ratio 50%.

We conduct results with 20% and 50% duplication. As duplication increases, the performance of our algorithm improves, as shown in Figure 8(b). In this simulation, for simplicity, we assume a number of tags up to 300. If number of tags is increased, difference will be more apparent. Due to this fact, our algorithm will be a better choice, especially in dense deployments. In Figure 9, we measured the computation cost of EIFS in comparison with INPFM and CLIF in terms of number of relays. INPFM filters all the data at every k hop node. The computation cost of CLIF in first round is the same as in our proposed scheme; however, in subsequent rounds it increases. Therefore, EIFS performs better with both 20% and 50% duplications and is a better choice especially in dense deployments in terms of communication and computation costs.

In the proposed scheme, if inter-cluster duplication is being filtered at neighboring nodes at certain hops called *α*, we don’t need to send feedback message and update routing table.

**Figure 10.**
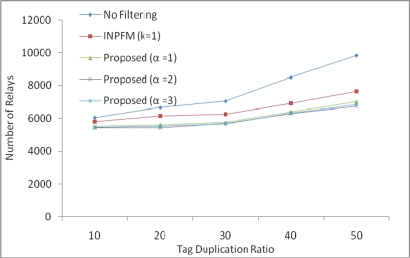
Number of relays with value of α.

We conducted a simulation to measure the suitable value of *α* in our environment. However, the value of *α* may vary with the environment. We measured the number of relays required to send data to a BS with different values of *α* by changing the duplication ratio of tags, as shown in Figure 10. With values of *α* as 1, 2, and 3, our scheme performs better than the EIRF, which is tree based approach. The best results occur when *α* is 2.

**Figure 11.**
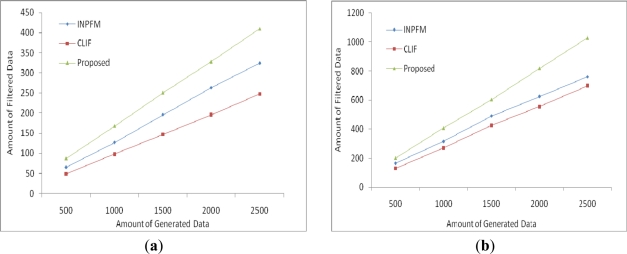
Amount of data filtered (**a**) 20% duplication (**b**) 50% duplication.

In above simulations, we proved that our proposed scheme saves more communication and computation cost compare to INPFM and CLIF. We can say our scheme is more energy efficient. We compared the filtering performance of all three approaches. It is clear that our proposed scheme filters more than 80% of duplications. Moreover, performance of our algorithm improves with the increased duplication ratio of tags, as shown in Figure 11.

In sensor network, maximum energy consumption happens when nodes transmit or receive data. Energy consumed in transmission and reception is much higher than energy spent in other tasks. EIFS saves a lot of communication overhead. Therefore, we can claim that EIFS is much more energy efficient than other in-network RFID data filtering schemes. In our future work, we plan to extend our simulation to measure energy consumption and network lifetime.

## 6. Conclusions

RFID is a revolutionary technology, but it does not support multi-hop communication which limits it to fewer applications. However, after integrating it with WSN, we can use WSN protocols for multi-hop communication of RFID data. In WSNs, energy is the most critical factor to be considered, whereas RFID data contains enormous amount of duplicate readings. Transmitting such duplicate data towards base stations wastes the nodes’ energy and results into decreased network lifetime. To save node energy, we need to filter this duplicate data within the network. In this paper, we propose Energy Efficient In-network RFID Data Filtering Scheme (EIFS) that divides the nodes into clusters. Every cluster head filters the data of its member nodes and send it towards the base station. Inter-cluster data is being filtered at neighboring nodes along the route. Our scheme filters the duplication close to source with less number of comparisons. Our work saves communication and computational cost and increases the network lifetime compare to other literature solutions. In our future works we will consider differential filtering where nodes will filter the amount of data considering their energy resources. In other words, every node will not eliminate all the duplications but the amount that is appropriate for to its energy resources. Filtering all the data within the network is not always an efficient solution.

## Figures and Tables

**Table 1.** Structure of the RFID data packet.

**Field**	**Tag *ID***	**Reader *ID***	**Time Stamp**	**Number of remaining filtering**
Byte	8	4	4	4

**Table 2.** Structure of the tag list stored in the cluster head.

**Field**	**Tag *ID***	**Reader *ID***	**Time Stamp**	**Observation *(β)***	**Redundant reader *ID(N)***
Byte	8	4	4	1	4

**Table 3.** Structure of 0intra-cluster node data packet.

**Tag *ID***	**Reader *ID***	**Time Stamp**	***f***
*x*	*N*	*T*	*1*

**Table 4.** Structure of inter-cluster node data packet.

**Tag *ID***	**Reader *ID***	**Time Stamp**	***F***
*x*	*n*	*t*	*f_e_*

**Table 5.** Simulation Environment.

**Parameters**	**Value**
Field Area	100 × 100 m^2^
Number of nodes	361
Number of clusters	19
Members in a cluster	19 (including cluster head)
Reading Range	5 m
Transmission Range	10 m
Reading interval	2 s
Duplication ratio	20% and 50%
